# Medical and Philosophical Intelligence

**Published:** 1815-06

**Authors:** 


### MEDICAL AND PHILOSOPHICAL INTELLIGENCE.

*To the Editors of the London Medical and Physical Journal.*

*Edinburgh Medical Hall, May* 22, 1815.

GENTLEMEN,

You will confer a favour on the Royal Medical Society of Edinburgh, by inserting in your valuable publication the following paragraph:—

The Royal Medical Society propose, as the subject of the Prize Essay for 1816, the following question:

"What changes of composition does the process of digestion in quadrupeds produce on earths, oxides, and earthy, alkaline, and metallic salts?"

A set of books, or a medal of five guineas value, shall be given annually to the author of the best Dissertation on an experimental subject proposed by the Society, for which all the members, honorary, extraordinary, and ordinary, shall alone be invited as candidates.

The Dissertations are to be written in English, French, or Latin, and to be delivered to the secretary on or before the 1st of December of the succeeding year to that in which the subjects are proposed; and the adjudication of the prize shall take place in the last week of February following.

To each Dissertation shall be prefixed a motto; and this motto is to be written on the outside of a sealed packet, containing the name and address of the author. No dissertation will be received with the author's name affixed; and all dissertations, except the successful one, will be returned, if desired, with the sealed packet unopened. Your most obedient servant,

NICOLSON BAIN, Secretary.

---

*Preternatural Labour.*—Professor W. C. BOWEN, of Providence, in a private letter to Dr. W. Channing, states the following case: "I was called to a woman who had been in labour between three and four days, and was nearly exhausted when I saw her. One arm of the child had presented, and *had been cut off*, by the attending surgeon before I was called. On examining the woman, I found the vagina completely filled, and the mutilated shoulder presenting at the external parts. With the crotchet, I first separated the shoulder blade, dislodged the contents of the thorax and abdomen, and then dislocated one of the dorsal vertebræ. All difficulty being now overcome, I easily turned and delivered the child. As this patient had great tension and tenderness of the abdomen and of the external parts when I first saw her, I was very apprehensive that extensive inflammation and sloughing would follow, but fortunately this has not been the case; the woman is in a fair way of recovery, and it is a fortnight since she was delivered." In another

 letter letter of a later date, in answer to some inquiries I made in my answer to his first, Dr. B. observes, "In the case lately described to you," viz. the above, "the left arm of the child presented, the head was towards the right side of the pelvis, and the breech and feet of course to the left, and towards the fundus uteri. It was physically impossible that *spontaneously evolution* could have taken place in this case, the child was so completely jammed into the vagina, and the uterus rigidly contracted upon it."

Dr. Denman, in his Aphorisms on the use of the Forceps and Vectis, treats concerning cases similar to the one above mentioned; but neither he nor any other author I have consulted, recommends the practice followed previous to Dr. Bowen's being called in for consultation. Hoornius recommends indeed, in such a case, to separate the head from the trunk, then turn the child, extract it, and then bring away the head. Heister's comment on this practice is full of cautions. We are to be certain that the child is dead, or that the mother is in the greatest danger from the longer continuance of ineffectual labour. We are advised by Dr. Denman, in the work above quoted, in a case similar to that just related, (viz.) where the arm and shoulder are completely jammed in the vagina, and the uterus firmly contracted on its contents, to wait for what he terms a spontaneous evolution of the child, by means of which the arm will be drawn up from the vagina, and the child be so altered in its presentation, as that the breech shall be first expelled.—*New England Journal.*

---

*Loss of the lower Jaw.*—In one of the late naval battles, a sailor had great part of the inferior jaw-bone carried off by a grape shot. The appearance of this poor man was frightful. His face was swelled, mouth wide open, and great qualities of saliva pouring out of it. In about 14 days, the shattered bones separated from the flesh, the swelling subsided, the mouth closed, so as to allow the patient to swallow and articulate. This favourable termination is to be attributed to the singular preservation of the under lip.—*New England Journal.*

---

*New Method of Treating the Itch.*—M. Ranque, Physician at Orleans, in France, has published a small book on the treatment of the itch, in which he proposes to lay aside the use of ointments and sulphureous medicines. His objections to the common mode are founded on the numerous ill consequences attributed to it by himself and other French physicians of reputation. He adopts that opinion of the nature of this disease, which has of late years become prevalent, viz.:—That it is produced by the propagation of an insect, *pediculus scabici.* This insect, he thinks, may penetrate to the internal parts of the body, and attack the great viscera. The application of powerful ointments to destroy this animal is supposed to interrupt the function of the skin and materially to impair its healthy action. M. Ranque states that, in the space of three years, three hundred Italians perished in the Hotel-Dieu of Orleans, solely from the effects of the itch. His medicine is prepared in the following manner:

Take, of powder of the grains of stavesacre, half an ounce, extract of the common poppy two drachms, boiled in a quart of water three quarters of an hour; do not express it; preserve it for use and strain it when employed. Sometimes, ten grains of the muriate of mercury are added. This decoction is applied warm in winter. It is to be rubbed thoroughly over the body three or four times a day, with a coarse linen rag, in such way as to break the pimples. It requires to be repeated from six to twelve days, successively. The author states that M. Corvisart, Larrey, Richerand, and others, think well of this method.

---

Mr. Wadd has lately opened the head of an epileptic subject, and found the left hemisphere of the brain entirely destroyed by suppuration. The patient, in the latter part of his life, was blind in the right eye. He retained his intellects to the last, and could sometimes express his wishes.

---

The operation for the popliteal aneurism has been performed in Dublin with a simplicity which does honour to the surgeon (Mr. Crampton), and confirms Mr. Jones's experiments. A single ligament was made, without incision, and withdrawn in a short time; in consequence of which the artery became obliterated, and the wound healed by the first intent.

---

Mr. Carpue has succeeded a second time in his Taliacotian operation, conducted on the Asiatic plan, greatly improved. The subjects were both military, and their improved physiognomy has been sufficient to attract the notice of the Commander-in-Chief, and even of the Prince Regent, who has appointed Mr. C. his Surgeon-Extraordinary.

---

The number of medical students at present in London is smaller than for several summers past.

---

Mr. Astley Cooper is preparing for re-publication his book on the Anatomy and Surgical Treatment of Hernia.

---

Mr. Wadd is preparing a most interesting work on Diseases of the Urinary Organs and Genitals. It is illustrated with engravings from his own drawings, and, if we are rightly informed, he has undertaken the etching also. If so, we may expect a degree of accuracy rarely to be met with in works of this description. The first part, on the Diseases of the Prostate Gland and Bladder, is already in the press.

---

Mr. Carpue will resume his Lectures on Anatomy and Surgery, as usual, the beginning of June, at his house in Dean-street, Soho.

---

Dr. Merriman's Lectures on Midwifery and the Diseases of Women and Children, will recommence on Monday, June 5th, at the Middlesex Hospital.

LONDON

### LONDON PRICES OF DRUGS.—May 26, 1815.

| | £. | s. | d. | £. | s. | d. | per |
|---|---|---|---|---|---|---|---|
| Aloes Barbadoes, from | 22 | 0 | 0 to | 0 | 0 | 0 | C. |
| —— Cape | 6 | 10 | 0 | 7 | 0 | 0 | — |
| —— Succotrina | 23 | 0 | 0 | 0 | 0 | 0 | — |
| —— Epatica or E.I. | 14 | 0 | 0 | 16 | 0 | 0 | — |
| Angelica Root | 8 | 0 | 0 | 8 | 10 | 0 | — |
| Alkanet Root | 8 | 10 | 0 | 0 | 0 | 0 | — |
| Antimony Crude | 2 | 18 | 0 | 3 | 5 | 0 | — |
| Aqua Fortis | S. | 0 | 8 | D. | 1 | 1 | lb. |
| Arrow Root, fine | 0 | 2 | 0 | 0 | 3 | 6 | — |
| —— ordinary | 0 | 0 | 9 | 0 | 1 | 3 | — |
| Arsenic, Red | 4 | 4 | 0 | 4 | 10 | 0 | C. |
| —— White | 2 | 16 | 0 | 0 | 0 | 0 | — |
| Balsam Capivi | 0 | 5 | 0 | 0 | 5 | 3 | lb. |
| —— Peru | 1 | 0 | 0 | 1 | 1 | 0 | — |
| —— Tolu | 0 | 14 | 6 | 0 | 15 | 0 | — |
| Bark Jesuits', Com. | 0 | 3 | 6 | 0 | 4 | 0 | — |
| —— Second | 0 | 6 | 0 | 0 | 7 | 0 | — |
| —— Quil or best | 0 | 8 | 0 | 0 | 10 | 0 | — |
| —— Red | 0 | 8 | 8 | 0 | 9 | 0 | — |
| —— Yellow | 0 | 2 | 0 | 0 | 2 | 2 | — |
| Borax, refined E.I. | none. | | | | | | |
| —— English | 0 | 2 | 8 | 0 | 3 | 0 | lb. |
| —— unrefined or Tinc. | 6 | 10 | 0 | 7 | 15 | 0 | C. |
| Camphire, refined | 0 | 6 | 0 | 0 | 6 | 3 | lb. |
| —— unrefined | 16 | 0 | 0 | 17 | 10 | 0 | C. |
| Cantharides | 0 | 11 | 0 | 0 | 0 | 0 | lb. |
| Cardamoms (best) | 0 | 6 | 6 | 0 | 7 | 6 | — |
| Cassia Buds | 32 | 0 | 0 } | 0 | 0 | 0 | C. |
| —— Fistula, W.I. | 5 | 5 | 0 } | in bond | | | — |
| —— Lignea | 42 | 0 | to } | 45 | 0 | 0 | — |
| Castoreum American | 2 | 10 | 0 | 3 | 0 | 0 | lb. |
| —— Russia | 11 | 11 | 0 | 0 | 0 | 0 | — |
| Castor Oil, per bottle 1½ lb. | 0 | 3 | 9 | 0 | 4 | 0 | bo |
| Cocculus Indicus | 6 | 0 | 0 | 7 | 0 | 0 | C. |
| Colocynth Turkey | 0 | 6 | 0 | 0 | 0 | 0 | lb. |
| Columbo Root | 4 | 15 | 0 | 6 | 0 | 0 | C. |
| Cream of Tartar | 6 | 0 | 0 | 6 | 10 | 0 | — |
| Gallangal East-India | uncertain | | | 0 | 0 | 0 | C. |
| Gentian Root | 3 | 10 | 0 | 3 | 15 | 0 | — |
| Ginseng | none | | | 0 | 0 | 0 | lb. |
| Grains of Guinea | 6 | 10 | 0 | 0 | 0 | 0 | C. |
| Gum Ammo. Drop | 50 | 0 | 0 | 0 | 0 | 0 | — |
| —— Lump | 5 | 0 | 0 | 7 | 0 | 0 | — |
| —— Animi | 3 | 10 | 0 | 7 | 10 | 0 | — |
| —— Arabic, E.I. | 2 | 0 | 0 | 4 | 0 | 0 | — |
| —— Turkey, Fine | 8 | 15 | 0 | 10 | 10 | 0 | — |
| —— Barbary | 5 | 5 | 0 | 0 | 0 | 0 | — |
| —— Assafœtida | 10 | 0 | 0 | 15 | 0 | 0 | — |
| —— Benjamin | 15 | 0 | 0 | 60 | 0 | 0 | — |
| —— Gambogium | 28 | 0 | 0 | 30 | 0 | 0 | — |
| —— Copal, scraped | 0 | 5 | 9 | 0 | 6 | 0 | lb. |
| —— Galbanum | 30 | 0 | 0 | 0 | 0 | 0 | C. |
| Gum Guaiacum, from | 0 | 2 | 6 to | 0 | 3 | 0 | lb. |
| —— Mastic | 0 | 4 | 3 | 0 | 4 | 6 | — |
| —— Myrrh | 15 | 0 | 0 | 18 | 0 | 0 | C. |
| —— Olibanum | 8 | 0 | 0 | 11 | 0 | 0 | — |
| —— Oppoponax | 80 | 0 | 0 | 0 | 0 | 0 | — |
| —— Sandrac | 8 | 0 | 0 | 8 | 5 | 0 | — |
| —— Senegal | 6 | 15 | 0 | 7 | 0 | 0 | — |
| —— Tragacanth | 27 | 0 | 0 | 27 | 10 | 0 | — |
| Jalap | 0 | 5 | 6 | 0 | 5 | 9 | lb. |
| Ipecacuanha | 0 | 14 | 6 | 0 | 15 | 0 | — |
| Isinglass Book | 0 | 9 | 0 | 0 | 10 | 0 | — |
| —— Leaf | 0 | 7 | 10 | 0 | 8 | 6 | — |
| —— Long Staple | 0 | 9 | 0 | 0 | 10 | 6 | — |
| —— Short Staple | 0 | 8 | 0 | 0 | 8 | 6 | — |
| Manna Flakey | 0 | 5 | 10 | 0 | 6 | 6 | — |
| —— Sicily in Sorts | 0 | 0 | 0 | 0 | 0 | 0 | — |
| Musk China | 0 | 14 | 0 | 0 | 16 | 0 | oz. |
| Nux Vomica | 2 | 0 | 0 | 2 | 2 | 0 | C. |
| Oil of Vitriol | 0 | 0 | 3¼ | 0 | 0 | 4 | lb. |
| Opium, East-India | none | | | 0 | 0 | 0 | — |
| —— Turkey | 1 | 4 | 6 | 0 | 0 | 0 | — |
| Pink Root | 0 | 4 | 0 | 0 | 4 | 6 | — |
| Quicksilver | 0 | 6 | 0 | 0 | 6 | 4 | — |
| Rhubarb, East-India | 0 | 9 | 0 | 0 | 13 | 0 | — |
| —— Russia | 0 | 18 | 0 | 1 | 0 | 0 | — |
| Saffron, Spanish | 2 | 12 | 0 | 2 | 16 | 0 | — |
| —— French | 1 | 18 | 0 | 2 | 0 | 0 | — |
| Sago | 6 | 15 | 0 | 8 | 10 | 0 | C. |
| Sal. Ammoniac | 10 | 10 | 0 | 11 | 0 | 0 | — |
| Sarsaparilla | 0 | 2 | 3 | 0 | 4 | 9 | lb. |
| Sassafras | 7 | 10 | 0 | 8 | 0 | 0 | T. |
| Scammony, Aleppo | 1 | 14 | 0 | 1 | 15 | 0 | lb. |
| —— Smyrna | 0 | 18 | 0 | 0 | 0 | 0 | — |
| Senna, Alexandria | 0 | 4 | 3 | 0 | 4 | 6 | — |
| Seeds, Anni Alicant | 4 | 15 | 0 | 5 | 0 | 0 | C. |
| —— Coriander English | 0 | 14 | 0 | 0 | 0 | 0 | — |
| —— Cummin | 5 | 1 | 0 | 0 | 0 | 0 | — |
| —— Fenugreek | 1 | 15 | 0 | 0 | 0 | 0 | — |
| Shellack | 7 | 10 | 0 | 10 | 15 | 0 | — |
| Sticklack | 3 | 10 | 0 | 7 | 0 | 0 | — |
| Snake Root | 0 | 15 | 6 | 0 | 0 | 0 | lb. |
| Soap, Castile or Spanish | 9 | 0 | 0 | 10 | 0 | 0 | C. |
| Spermaceti, refined | 0 | 2 | 2 | 0 | 0 | 0 | lb. |
| Tamarinds, West-India | 9 | 0 | 0 | 10 | 0 | 0 | C. |
| Tapioca, Lisbon | 0 | 0 | 9 | 0 | 1 | 0 | lb. |
| Turmeric, Bengal | 4 | 10 | 0 | 4 | 15 | 0 | C. |
| —— China | 6 | 10 | 0 | 7 | 0 | 0 | — |
| —— West-India | 3 | 10 | 0 | 4 | 4 | 0 | — |
| Verdigris, Wet | 0 | 3 | 6 | 0 | 0 | 0 | lb. |
| —— Dry | 0 | 7 | 0 | 0 | 7 | 6 | — |
| —— Crystallized | 0 | 9 | 0 | 0 | 9 | 6 | — |
| Vitriol, Roman | 0 | 0 | 7 | 0 | 0 | 0 | — |
| —— Foreign white | 2 | 10 | 0 | 0 | 0 | 0 | C. |

*Price of Vials per Gross.*—8 oz. 70s.—6 oz. 58s.—4 oz. 47s.—3 oz. 43s.—2 oz. 36s.—1 oz. 30s. —½ oz. 24s.

Lisbon Leeches, 30s. per hundred.—True French ditto, 55s.

Essential Salt of Lemons, 4s. 6d. per dozen.

### METEOROLOGICAL REGISTER.

*From April the 25th, to May the 25th,* 1815.

Kept by C. BLUNT, Philosophical Instrument Maker, No. 38, Tavistock-Street, Covent-Garden.

| Moon. | Day. | Wind. | Barometrical Pressure. Max. | Min. | Mean. | Temperature. Max. | Min. | Mean. | |
|---|---|---|---|---|---|---|---|---|---|
| | 26 | N | 30·06 | 29·93 | 30·032 | 55 | 36 | 45·5 | Rain |
| | 27 | NW | 30·03 | 29·97 | 29·99 | 57 | 37 | 47 | Fair |
| | 28 | NW | 29·99 | 29·80 | 29·887 | 57 | 38 | 47·5 | Fair |
| | 29 | W | 29·70 | 29·60 | 29·645 | 59 | 39 | 49 | Fair |
| | 30 | W | 29·67 | 29·67 | 29·67 | 62 | 40 | 51 | Fair |
| ◑ | 1 | NW | 29·73 | 29·70 | 29·722 | 65 | 45 | 55 | Fair |
| | 2 | NNW | 29·78 | 29·74 | 29·755 | 68 | 50 | 59 | Fair |
| | 3 | W | 29·73 | 29·69 | 29·71 | 70 | 51 | 60·5 | Fair |
| | 4 | SW | 29·75 | 29·73 | 29·735 | 70 | 50 | 60 | Fair |
| | 5 | SW | 29·74 | 29·72 | 29·73 | 70 | 52 | 61 | Rain |
| | 6 | S | 29·75 | 29·75 | 29·75 | 74 | 50 | 62 | Rain |
| | 7 | S | 29·74 | 29·74 | 29·74 | 75 | 51 | 63 | Fair |
| | 8 | SE | 29·73 | 29·70 | 29·72 | 72 | 49 | 60·5 | Fair |
| ● | 9 | SE | 29·94 | 29·88 | 29·92 | 75 | 51 | 63 | Fair |
| | 10 | S | 29·94 | 29·90 | 29·922 | 76 | 50 | 63 | Fair |
| | 11 | S | 29·83 | 29·66 | 29·747 | 77 | 55 | 66 | Fair, Thunder storm at 9 p.m. |
| | 12 | SW | 29·74 | 29·67 | 29·697 | 71 | 50 | 60·5 | Rain |
| | 13 | W | 29·76 | 29·71 | 29·722 | 71 | 48 | 59·5 | Rain |
| | 14 | W by S | 29·80 | 29·80 | 29·80 | 70 | 48 | 59 | Rain |
| | 15 | SW | 29·82 | 29·82 | 29·82 | 70 | 50 | 60 | Fair |
| ◐ | 16 | SW | 30·15 | 29·99 | 30·087 | 75 | 51 | 63 | Fair |
| | 17 | W | 30·28 | 30·25 | 30·26 | 73 | 50 | 61·5 | Fair |
| | 18 | W | 30·23 | 30·17 | 30·205 | 70 | 52 | 61 | Fair |
| | 19 | W | 30·16 | 29·94 | 30·045 | 69 | 52 | 60·5 | Fair |
| | 20 | W by N | 29·82 | 29·66 | 29·722 | 66 | 47 | 56·5 | Fair |
| | 21 | NW | 29·71 | 29·71 | 29·71 | 65 | 50 | 57·5 | Rain |
| | 22 | NW | 29·83 | 29·91 | 29·873 | 61 | 50 | 55·5 | Fair |
| ○ | 23 | W | 29·91 | 29·77 | 29·83 | 61 | 49 | 55 | Rain |
| | 24 | W | 29·91 | 29·84 | 29·87 | 64 | 50 | 57 | Rain |
| | 25 | W | 30·06 | 30·05 | 30·055 | 65 | 50 | 57·5 | Fair |

RESULTS.

| | | | | | |
|---|---|---|---|---|---|
| Mean barometrical pressure of the month | | 29·845 | Mean temperature of the month | | 57·9 deg. |
| Maximum 30·28 | wind at | W | Maximum 77, | wind at | S |
| Minimum 29·60 | ——— | W | Minimum 36, | ——— | N |

*Scale exhibiting the prevailing Winds during the Month.*

| N | NE | E | SE | S | SW | W | NW |
|---|---|---|---|---|---|---|---|
| 1 | 0 | 0 | 2 | 4 | 5 | 12 | 6 |

| | Mean barometrical pressure. | Mean temperature. |
|---|---|---|
| From the last quarter on the 1st May, to the new moon on the 9th | 29·732 | 50·1 |
| ——— new moon on the 9th, to the first quarter on the 16th | 29·804 | 61·5 |
| ——— first quarter on the 16th, to the new moon on the 23d | 29·936 | 59·3 |

REPORT

### REPORT OF DISEASES.

The discases still continue to assume an inflammatory form. Those who have passed the late mild winter with an alleviation of their customary pulmonary symptoms, have, many of them, in the spring, been assailed with hæmoptysis, or acute inflammation. The former has, for the most part, yielded to purgative medicines. In some instances blood-letting has been added. None of them seem to threaten mischief excepting a single instance of a young female, native of a warmer climate, who found all her complaints alleviated during the winter, and who, since the return of spring, has, besides hæmoptysis, a quick pulse, flushed cheek, short cough, with hurried respiration. Some embarrassments, in consequence of her new situation in a foreign country, have probably exasperated all these symptoms, which, however, in an interesting young female, cannot be contemplated without the anxiety of her friends.

A case of epileptic fits has occurred in a stout lad, about the age of puberty: it has given way to strong and repeated purgatives.

Several instances of apoplexy, and consequent paralysis, in their various stages and forms, have occurred; some of the former with symptoms of relapse. All these have been relieved with venesection or cupping. Cupping has been used with great freedom, and almost always with advantage. The general remark, that blood taken in this way induces less debility than when taken from the vein, is daily proved, and may probably be explained by reflecting that a considerable part of the fluid may consist of different juices, as well as blood, which may account for the imperfect coagulation. The other cases are much assisted by frequent purgatives, which the patients bear extremely well, whilst the appetite remains good.

Rheumatism has almost disappeared. Even the occurrence during the temporary cold weather was inconsiderable, and easily managed.

A case of diabetes mellitus has occurred in a youth about the age of puberty. It was attended with symptoms bordering on idiotism, and so refractory did the patient prove, that little could be learned concerning the effect of, or even the manner in which the prescribed remedies were taken. The confining him to animal food was, however, accomplished long enough to produce a change in the properties, though not in the quantity, of the urine.

Several cases of typhus mitior have occurred, without any dangerous symptoms: congestion about the head, and profuse perspiration, without proportionate relief, have been the principal symptoms.

